# Effect of the aqueous extract of *Senecio biafrae *(Oliv. & Hiern) J. Moore on sexual maturation of immature female rat

**DOI:** 10.1186/1472-6882-12-36

**Published:** 2012-04-06

**Authors:** Landry L Lienou, Bruno P Telefo, Bayala Bale, Didiane Yemele, Richard S Tagne, Stephanie C Goka, Chantal M Lemfack, Celestin Mouokeu, Paul F Moundipa

**Affiliations:** 1 University of Dschang, Faculty of Science, Department of Biochemistry, P.O Box: 67 Dschang, Cameroon; 2 University of Ouagadougou, UFR/SVT, Laboratory of Animal Physiology, 03 P.O. Box 7021, Ouagadougou 03 Burkina Faso, Africa; 3 University of Yaounde I, Faculty of Science, Department of Biochemistry, P.O Box: 812, Yaounde, Cameroon

## Abstract

**Background:**

*Senecio biafrae *(Asteraceae) is a medicinal plant widely used by traditional healers in the western region of Cameroon for the treatment of female infertility. This experiment was designed to evaluate the effect of the aqueous extract from leaves and stems of *S. biafrae *(AESb) on the onset of puberty and some biochemical and physiological parameters of reproduction in immature Wistar female rats.

**Methods:**

Different doses of AESb were daily and orally administered to immature female rats (13 animals/group) for 30 days. At the end of the treatment period, six animal of each experimental group were sacrificed and their body, ovarian, uterus weight; uterine, ovarian protein or cholesterol level as well as data on puberty onset recorded. The remaining animals of each group were used for the fertility test and some gestational parameters recorded.

**Results:**

A linear increase in the growth rate of all animals was observed. The body weight gain in animals treated at the dose of 8 mg/kg of AESb significantly increased (p < 0.05) after 25 days of treatment while those receiving the doses of 32 and 64 mg/kg presented a significantly low body weight gain starting from the 19^th ^day till the end of the treatment period. The ages (days) of animals at vaginal opening (VO) was significantly reduced (p < 0.05) in those treated with the doses of 32 (41.25 ± 0.51) and 64 mg/kg (41.42 ± 0.54) as compared to control animals (43.33 ± 0.73). AESb significantly increased (p < 0.05) the ovarian weight and the number of corpora lutea in animals treated with 8 mg/kg as well as the uterine weight and protein levels irrespective of the dose. No significant effect of the extract on various fertility and gestational parameters was registered.

**Conclusion:**

The overall results of the present study provide evidence on the puberty onset induction and ovarian folliculogenesis effect of AESb in immature female rat.

## Background

Medicinal Plants have been used for many years in daily life to treat diseases all over the world [1,2]. According to the World Health Organization (WHO), more than three-quarters of the world's population rely upon complementary and alternative medicine for health care [3]. Many medicinal plants are used to treat various reproductive function ailments such as female infertility which is a public health concern in Sub-Saharan African countries [4]. *Senecio biafrae *(Oliv. & Hiern) J. Moore (Asteraceae) (syns. *Crassocephalum biafrae *and *Solanecio biafrae*) is one of these plants [5]. It is a perennial climbing herb which naturally occurs in African forest zones, from Guinea to Uganda. Its leaves contain various secondary metabolites such as dihydroisocoumarins, terpenoids, sesquiterpenes or amino acids [6-8]. *Senecio biafrae *is equally known for its therapeutic virtues, notably in Nigeria where it is used in the treatment of diabetes or pulmonary defects [9-11]. In Benin, Côte d'Ivoire, Congo, or Cameroon it is used in traditional medicine to treat many diseases such as bleeding from cuts, sore eyes, cough, heart troubles, rheumatic pain, or localized oedemas [6]. In the Western and North-western Regions of Cameroon, ethnobotanical studies revealed its utilization in the treatment of cases of women infertility [5,12].

Many conditions can be associated to infertility, among which non avoidable factors (anatomic, genetic, hormonal and immunological problems) and avoidable factors such as Sexually Transmitted Infections (STI), infections after parturition or surgery, tuberculosis of the pelvis, and obesity [13,14]. A range of medical and alternative treatments exist for infertility. They include the use of fertility drugs to stimulate "superovulation", intrauterine insemination, Assisted Reproductive Technologies (ARTs) and medicinal plants preparation to which most of the infertile couple in developing countries generally rely for their treatment [15,16].

Many studies have indicated the implication of secondary metabolites from medicinal plants on the regulation of reproductive functions [17-21]. These metabolites act on main organs of the reproductive system to inhibit or induce ovarian folliculogenesis. Their biological activities are often evaluated on reproductive organs of immature female rats which have long been used as a model system for studying, *in vivo*, the inducing effect of pharmacological compounds [22,23] and medicinal plants [18,24,25] on ovarian folliculogenesis. In those various studies, the gonadotrophic-like effects of the preparation was characterized by the following biological parameters: increase in the weight of the ovary and uterus; opening and cornification of the vagina; formation of corpora lutea or changes in the histology of the ovary, uterus and vagina; induction of ovulation; increase in ovarian estradiol, progesterone, protein levels; decrease in ovarian cholesterol level.

The leaves and stems of *Senecio biafrae *are used either macerated in water or in palm wine by traditional healers of our locality to treat cases of women infertility [5]. Our previous studies on the effect of its ethanolic extract on some physiological parameters of reproduction showed an increase in ovarian or uteri weights and proteins as well as an acceleration of the onset of puberty in immature female rats [26]. The present study therefore aimed at evaluating the effect of the aqueous extract of *S. biafrae *(AESb) on similar parameters of sexual maturation (puberty onset, fertility induction) in immature female rat. The precocity of the puberty onset in treated animals was evaluated through the determination of their age at vaginal opening and the inducing effect of the extract on animal fertility evaluated through the determination of ovarian and uterine weights, protein or cholesterol levels; number of corpora lutea, implantation sites and other gestational parameters.

## Methods

### Preparation of the extract

The fresh leaves and stems of *S. biafrae *were collected in May 2010 in Baham subdivision (Western Region of Cameroon) and identified at the National Herbarium of Cameroon under voucher specimen code 32999/SRF/Cam. These plant parts were washed and dried at room temperature. The dried plants were ground in a mortar and the powder obtained used to prepare the aqueous extract by maceration in distilled water for 24 h. At the end of this period, the solution was filtered before being dried in a ventilated oven at 45°C. The dry powder was weighed and stored at 4°C in a refrigerator. The yield of extraction was 16.6%. The extracts were prepared in distilled water at concentrations of 2.8 mg/ml (extract 1), 11.2 mg/ml (extract 2) and 22.4 mg/ml (extract 3). Together with distilled water (control group), these preparations (extracts 1, 2 and 3) were orally administered to animals in a volume of 3 ml/kg body weight, corresponding to doses of 0, 8, 32, and 64 mg/kg respectively. The dose of 8 mg/kg was reconstituted on the basis of information from traditional medicine practitioners in an ethnopharmacological survey performed in Baham subdivision (Western Region of Cameroon), and the two other doses (32 and 64 mg/kg) were its multiples

### Animals

The animals used in this study were immature female albino Wistar rats, 21-22 days old, weighing 30-45 g. They were bred in the animal house of the Biochemistry Department (University of Dschang, Cameroon), housed under natural conditions of light (12 h cycle) and temperature (22 ± 2°C) and fed with a standard laboratory diet and tap water *ad libitum*.

#### Ethical consideration

Experimental protocols used in this study strictly conformed with the internationally accepted standard ethical guidelines for laboratory animal use and care as described in the European Community guidelines; EEC Directive 86/609/EEC, of the 24th November 1986 [27].

### Puberty onset and fertility assays

A total of fifty-two immature female rats were randomized, based on their body weight, into 4 groups of thirteen animals each. They received by gavage, either distilled water or different doses of AESb for 30 consecutive days. Their body weight and food intake were recorded, throughout the experimental period, at 2 day intervals. After two weeks of treatment, each rat was checked every day for vaginal opening and the vaginal smear collected and stained from the day of opening up to the end of the experiment. The vaginal smears were stained by May-Grünwald solution (1%w/v) followed by Giemsa solution (1%w/v) and viewed under low magnification (40 ×) under a light microscope. This staining helped in characterizing each phase of the rat's estrous cycle, its length and that of the complete cycle. At the end of the experimental period, 6 animals in each group were randomly sacrificed by anesthesia using chloroform. Their ovaries and uteri were removed, blotted, weighed and stored at - 20°C until use.

The remaining rats (7 per group) were crossed the following day, during two weeks, with males of proven fertility. Vaginal smears were collected on a daily basis in order to assess for the presence of sperm. A laparoscopy was undertaken under diazepam (5 mg/ml, 5 mg/kg) and Ketamin (50 mg/ml, 80 mg/kg) ten days after the day of mating to count the number of implantation sites in uterine cords and the number of corpora lutea in ovaries. After delivery, the fetuses were weighed and their number recorded. From these data, the number of resorption sites (number of implantation site - number of live fetuses), implantation index ([total number of implantation sites/number corpora lutea] × 100), resorption index ([total number of resorption sites/total number of implantation sites] × 100), preimplantation loss ([number of corpora lutea - number of implantations/number of corpora lutea] × 100), postimplantation loss ([number of implantations × number of life fetuses/number of implantations] × 100), antifertility activity ([number of females without life fetuses/total number of females] × 100), antiimplantation activity ([number of females without implantation sites/total number of females] × 100), and gestation rate ([number of females with life fetuses at birth/total number of gestational females] × 100) were calculated [28].

### Preparation of the uterine and ovarian supernatants and biochemical analysis

Ovaries and uteri were homogenized in Tris - sucrose buffer (0.25 M sucrose, 1 mM EDTA and 10 mM Tris-HCl, pH 7.4) at 1% and 2% respectively. The homogenate was then centrifuged at 6000 × *g *at 4°C (Beckman model J2-21) for 15 min, and the supernatants collected were used for protein [29] and cholesterol [30-32] assays.

### Statistical analysis

The data from biological assays were treated by the one way Analysis of Variance (ANOVA) test and presented as Mean ± SEM (standard error on the mean). The Fisher PLSD test was used for the comparison of means. The analysis of percentages was done by X^2 ^(Chi-square) test. The Kruskall-Wallis test was used for non parametrical data, and the Mann Whitney test used when their differences were significant [33].

## Results

### Effect of AESb on body weight gain and food intake

**Figure 1:**
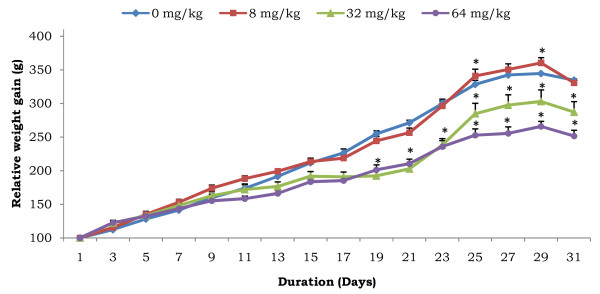
**Body weight gain of rats after administration of various doses of AESb**. Each point represents mean ± SEM of 13 animals per group. *Values statistically different from that of the control group of each day at P < 0.05 (ANOVA and Fisher PLSD).

**Figure 2:**
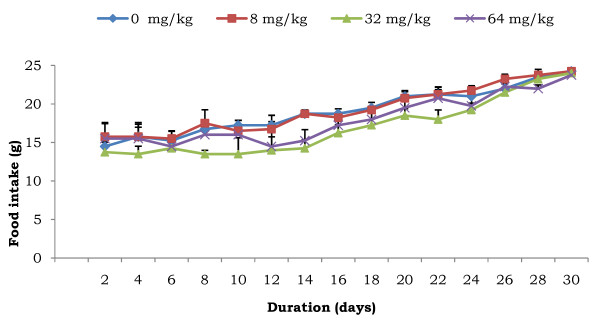
**Food intake of animals in function of the duration of treatment with various doses of AESb**. Each point represents mean ± SEM of 13 animals per group.

The effect of AESb on the body weight of female rats during the treatment is presented in Figure 1. There was a linear increase, at various rates, in their growth. Compared to control animals, a significant drop in the body weight of animals treated with the doses of 32 and 64 mg/kg was indeed noticed after 19 days of treatment. Animals receiving the 8 mg/kg dose instead gained more weight (p < 0.05) compared to control animals, starting from the 25th day of treatment till the end of the experiment. As concerns the monitoring of their food intake, no significant variation, whatever the duration of the treatment, was observed between the different experimental groups (Figure 2).

### Effect of AESb on the age and estrous cycle phases at vaginal opening

**Figure 3:**
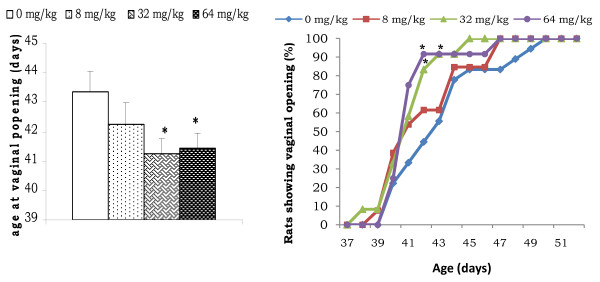
**Effect of prepubertal oral administration of AESb on the timing of Puberty onset**. Shown on the *left *are mean day of vaginal opening in control and prepubertal AESb-treated animals. Shown on the *right *are percentages of animal showing vaginal opening as a function of age in the control and AESb-treated animals. *Values significantly different at p < 0.05 from those of the control group (Kruskal-Wallis followed by Mann-Whitney tests for histograms and Khi Square followed by Fisher Exact tests for lines); each data represents the mean ± SEM of 13 animals.

Figure 3 shows the mean age of animals at vaginal opening and the percentage of those presenting vaginal aperture at a given age. Female rats that received AESb at the two highest doses presented vaginal opening almost two days earlier (p < 0.05) as compared to the control animals [43.33 ± 0.73 days (0 mg/kg) *vs *41.25 ± 0.51 days (32 mg/kg) or 41.42 ± 0.54 days (64 mg/kg)]. About 8% of 38 days old rats treated at 32 mg/kg (against 0% for its respective control) presented vaginal aperture. This percentage was significantly increased in these animals and in those treated at 64 mg/kg dose when they were 42 or 43 days old (92% with the two doses compared to 48% with 0 mg/kg). Moreover, complete vaginal opening was obtained in all animals of these experimental groups (32 and 64 mg/kg) when they were 45 or 46 days old, contrary to those of the control group for which it was obtained at the age of 50. Most of these animals presented an estrus (23% - 46%) or metoestrus (8%-46%) vaginal cycle phase on the day of their vaginal opening (Table 1).

**Table 1:** Frequency (%) of estrous cycle phases at vaginal opening in female rats treated with various doses of AESb

	Cycle phases				Total
**Doses**	**Proestrus**	**Œstrus**	**Metoestrus**	**Diestrus**	

**0 mg/kg**	19.05	42.9	33.3	4.8	100

**8 mg/kg**	23.1	46.2	7.7	23.1	100

**32 mg/kg**	23.1	23.1	46.2	7.7	100

**64 mg/kg**	38.5	38.5	15.4	7.7	100

(Fisher exact and Khi square tests).

### Effect of AESb on the estrous cycle length

No changes in the estrous cycle length of animals of various experimental groups as well as in the duration of the estrus, metestrus and diestrus phases of their cycle were observed (Table 2). However, a slight decrease in the duration of the proestrus phase of animals treated at the dose of 8 and 32 mg/kg was recorded.

**Table 2:** Effect of AESb on the duration (days) of the various phases in the estrous cycle

Doses	Phases of the estrous cycle				
	
	Proestrus	Estrus	Metestrus	Diestrus	Cycle Length
0 mg/kg	0.80 ± 0.17	1.38 ± 0.19	0.57 ± 0.11	1.22 ± 0.16	4.00 ± 0.16

8 mg/kg	0.56 ± 0.10	1.60 ± 0.19	0.57 ± 0.12	1.27 ± 0.15	4.00 ± 0.11

32 mg/kg	0.65 ± 0.13	1.58 ± 0.24	0.35 ± 0.07	1.42 ± 0.17	4.00 ± 0.19

64 mg/kg	0.75 ± 0.17	1.25 ± 0.19	0.73 ± 0.14	1.28 ± 0.11	4.01 ± 0.15

The length of the phases is expressed as days. Each value represents the mean ± SEM for 13 animals

### Effect of AESb on ovarian weight, protein or cholesterol level and corpora lutea number

**Figure 4:**
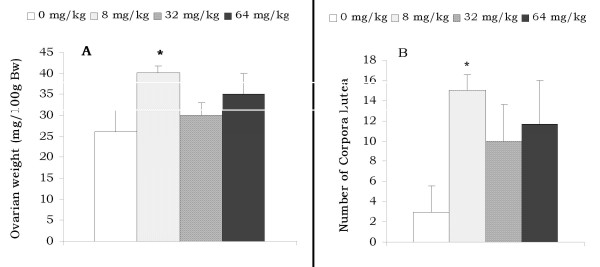
**Effect of AESb on the relative weight of the ovaries (A) and the number of corpora lutea (B)**. *Values significantly different at (p < 0.05) from those of the control group (ANOVA and Fisher PLSD). Each histogram represents the mean ± SEM of the values for 6 animals.

The changes obtained in ovaries after 30 days of oral administration of various doses of AESb to immature rats are presented in Figure 4. No significant variations in ovarian cholesterol and protein levels were recorded (data not shown). When administered at the dose of 8 mg/kg and in comparison to the control group, the extract significantly increased (P < 0.05) the number of corpora lutea, and the relative weight of ovaries (Figure 4A and 4B).

### Effect of AESb on uterine weight and proteins

**Figure 5:**
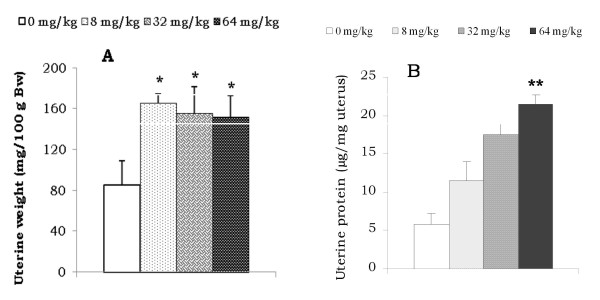
**Effect of AESb on uterine relative weight (A) and uterine proteins level (B)**. *Values significantly different at (p < 0.05) from those of the control group (ANOVA and Fisher PLSD); each histogram represents the mean ± SEM of the values for 6 animals.

Oral administration of AESb to immature female rats significantly increased the weight of their uteri irrespective the dose administered (Figure 5A). Uterine proteins were also increased in these animals notably by two (8 mg/kg) to three fold (32 and 64 mg/kg; P < 0.01) (Figure 5B).

### Effect of AESb on some fertility and gestational parameters

Table 3 shows that oral administration of AESb at doses of 8, 32 and 64 mg/kg showed no significant effect on the number of pregnant rats, implantation sites and fetal weight. However, a slight increase in the number of resorption sites at the dose of 64 mg/kg was obtained. This resulted in antiimplantation and antifertility activities of 14% and 29% respectively.

**Table 3:** Effect of AESb on some fertility and gestational parameters

Studied parameters		Dosage (mg/kg/Day)		
	
	0	8	32	64
N°Corpora Lutea	11.9 ± 0.31	11.57 ± 0.61	11.43 ± 0.90	10.33 ± 1.50

N°Implantation sites	10.80 ± 0.44	10.43 ± 0.57	10.43 ± 0.75	9.00 ± 1.48

N° Fetuses Alive	9.50 ± 0.50	9.29 ± 0.52	9.86 ± 0.77	7.83 ± 1.68

N°Resorption sites	1.30 ± 0.47	1.00 ± 0.44	0.57 ± 0.43	1.17 ± 0.48

Mean weight of fetuses (g)	4.87 ± 0.09	5.16 ± 0.17	5.24 ± 0.28	5.27 ± 0.13

Fixation rate (%)	90.93 ± 3.36	91.10 ± 5.15	91.77 ± 1.72	84.58 ± 5.06

Preimplantation Loss (%)	9.07 ± 3.36	8.90 ± 5.15	8.23 ± 1.72	15.42 ± 5.06

Postimplantation Loss (%)	0.12 ± 0.04	0.11 ± 0.04	0.05 ± 0.04	0.25 ± 0.16

Antiimplantation Activity (%)	0	0	0	14.29

Antifertility Activity (%)	0	0	0	28.57

Resorption Index (%)	12.037	9.589	5.480	12.963

Gestation Rate (%)	100	100	100	85.71

Each value represents the mean ± SEM for 7 animals.

## Discussion

*Senecio biafrae*, which is the plant of interest in this study, is used by some people in Africa for its nutritional and pharmacological properties [6,7,9-11]. Its effect on the onset of puberty (age and phase of the estrous cycle at vaginal opening) and the ovarian folliculogenesis of immature female rats were evaluated. The choice of these parameters was not only guided by the influence of the gonadotrophic hormones (FSH, LH, PMSG, GnRH) on the precocious onset of puberty and the induction of the follicular growth in immature female rats, but also by the clinical usage of these hormones in the treatment of various forms of infertility (ovulatory defects or hypogonadal infertility) [34]. Puberty is the culmination of a complete sequence of maturational events that lead to the activation of the gonadotrophic axis, linked to the increase in serum levels of LH and FSH and attainment of sexual maturity. Estradiol has been reported to peak just before the vaginal opening in female rats, showing that it is the effective molecule for puberty induction [35]. In various mammals, precocious puberty onset can be induced in a prepubertal animal by repeated injections of GnRH, FSH, LH or of an analogous compound of a general excitatory neurotransmitter of the central nervous system, like glutamate or aspartate which induce the pulsatile releases of GnRH [36]. The pulsatile secretion of GnRH hormone at puberty leads to the pulsatile release of FSH and LH. These pituitary hormones in turn enhance the proliferation of the follicular cells and the production of estrogens (principally estradiol) by ovarian cholesterol catabolism [37]. The overall hormonal signalization culminates in the opening and cornification of the vagina, and to the increase in ovarian and uterine weight [38].

Oral administration of AESb for 30 days, at doses of 32 and 64 mg/kg, to immature female rats led to a precocious onset of vaginal opening. This shows that the plant extract could contain molecules acting, as one of the above compounds, on the precocious onset of puberty. The same result was obtained, at 8 mg/kg dose, with the ethanolic extract of the plant [26]. This shows that the active compound of the plant would be more extracted by organic solvents.

The opening of the vagina on attainment of the pubertal age of rats resulted from the increase in the secretion of estrogens by ovarian follicles. The vaginal cells are also keratinized in this high estrogenic environment [39]. That is why the vaginal smear of pubertal rats the day of vaginal opening corresponds to the estrus phase of the cycle or to the nearest phase following it (metoestrus), as shown in Table 1. In animals treated with 8 and 32 mg/kg doses, the proestrus phase (preovulatory phase) was slightly reduced. This could be linked to the acceleration in the ovarian follicular growth, given the high estrogenic environment of the ovarian cells, following the induction of pulsatile secretion of GnRH and gonadotrophins by AESb.

A significant reduction in the body weight gain of animals, after three weeks of oral administration of AESB, was also noticed. During the same period of treatment, no significant changes in various biochemical parameters of toxicity (data not shown) nor in the daily food intake of AESB treated animals was observed. Thus, this body weight reduction may be related to the cumulated slight decrease, during the same period of treatment, in food intake of those treated with the doses of 32 or 64 mg/kg of AESB.

Estrogens and estrogen-like compounds (phytoestrogens) are well known regulators of growth and differentiation in a number of tissues. They exert their biological effect following their fixation to the receptors in their main target organs (ovary, uterus, hypothalamus, bone,...) thus leading to a chain of reactions, culminating in the biosynthesis of biomacromolecules (DNA, RNA, and proteins) and the increase in the weight of these organs, principally the ovary and uterus [1,40,41]. This weight increase is a combination of hypertrophic response of tissues following cell proliferative activities and water imbibition in the tissue [42]. The data on uterine parameters presented in this study has shown an increase of more than 96% and 87% in uterine proteins and uterine weight respectively, at all the doses. This increase in uterine proteins and weight may have resulted not only from the uterine cell proliferation induced by the oestrogenic effects of some chemical components of AESB but also from the increase in their water imbibition effect, especially at low dosage, in these cells. This estrogenic potential of AESB is also attested with the increase, at the dose of 8 mg/kg, in ovarian weight and corpora lutea number.

As concerns the test on gestational parameters, a slight reduction in the number of corpora lutea, and gestational rate with the highest dose (64 mg/kg) could be linked to the reduction in the ovulation rate of these animals following the negative feed-back effect, at the level of the hypothalamus, of high estrogen level induced by this dose of the extract [37]. This could also results from the fact that estrogenic compounds cause contraction in the uterine smooth muscle, which could lead to the resorption of the fetus after its implantation [43].

## Conclusion

Globally, this study has proven the implication of some compounds present in AESb on the rapid maturation of ovarian follicular cells leading to a precocious puberty onset. The mechanisms of this stimulation are multiple (induction of GnRH synthesis or secretion, induction of gonadotrophin synthesis or secretion, effects of estrogens-like compounds or specific amino acids present in the extract). Further studies are required for the elucidation of these mechanisms.

These results also corroborate with those obtained with the ethanolic extract of *S. biafrae*, but with different dosages. Thus, AESb would be less active than the ethanolic extract of the plant in inducing precocious puberty in immature female rats, but more safer for gestation. The therapeutic dose used by traditional medicine practitioners is able to stimulate folliculogenesis, but have a little effect on the maturation of the reproductive axis; for a better effect, this therapeutic dose should be increased. Unfortunately, high doses after 30 days of treatment exhibit adverse effects on gestational parameters. So, the length of the treatment with these doses should be shortened.

## Abbreviations

AESb: Aqueous extract of *Senecio biafrae*; GnRH: Gonadotropin Releasing Hormone; LH: Luteinizing Hormone; FSH: Follicle Stimulating Hormone; PMSG: Pregnant Mare Serum Gonadotropin; DNA: DeoxyriboNucleic Acid; RNA: RiboNucleic Acid; STI: Sexually Transmitted Infections; ARTs: Assisted Reproductive Technologies; WHO: World Health Organization.

## Competing interests

The authors declare that they have no competing interests.

## Authors' contributions

LLL, TSR and YMD collected the samples, prepared the extract and carried out all the animal model experiments. GSC, MC and LMC participated to the study design and the statistical analysis or interpretation of the data. BBR helped in manuscript drafting, discussion and revision. TPB and MFP supervised, evaluated the data and corrected the manuscript for publication. All authors read and approved the final manuscript.

## Pre-publication history

The pre-publication history for this paper can be accessed here:

http://www.biomedcentral.com/1472-6882/12/36/prepub
